# Optic Coherence Tomography for Accommodation Control in Children with Hyperopic Anisometropia and Amblyopia

**DOI:** 10.17691/stm2023.15.5.03

**Published:** 2023-10-30

**Authors:** I.L. Kulikova, K.A. Aleksandrova

**Affiliations:** MD, DSc, Deputy Director for Clinical Work; Cheboksary Branch of S. Fyodorov Eye Microsurgery Federal State Institution of the Ministry of Health of Russia, 10 Traktorostroiteley St., Cheboksary, 428000, Chuvash Republic, Russia; Professor of the Ophthalmology Course; Institute for Advanced Training of Doctors of the Ministry of Health of the Chuvash Republic, 27 Mikhail Sespel St., Cheboksary, 428018, Chuvash Republic, Russia; Ophthalmologist, Diagnostic and Treatment Department; Cheboksary Branch of S. Fyodorov Eye Microsurgery Federal State Institution of the Ministry of Health of Russia, 10 Traktorostroiteley St., Cheboksary, 428000, Chuvash Republic, Russia

**Keywords:** optical coherence tomography, ciliary muscle, anisometropia, amblyopia, accommodation

## Abstract

**The aim** is to evaluate the ocular accommodation system in hyperopic anisometropia and amblyopia in children after femtosecond laser-assisted *in situ* keratomileusis (FS-LASIK) and in children with spectacle correction using optical coherence tomography (OCT).

**Materials and Methods:**

The present study included children with hyperopia and anisometropia of more than 3 D, high and medium degree of amblyopia. Patients were divided into two groups: group 1 consisted of 30 children after FS-LASIK, group 2 was comprised of 30 children with spectacle correction. The temporal part of the ciliary muscle was assessed using the CASIA2 optical coherence tomography system (Tomey, Japan). The study was carried out with a narrow pupil fixing the gaze on the target at a distance of 33 cm and under cycloplegic conditions. The ciliary muscle thickness (CMT) was analyzed at four different levels: the maximum thickness of the ciliary muscle (CMT_max_), and at a distance of 1, 2, and 3 mm from the scleral spur (CMT_1_, CMT_2_, and CMT_3_, respectively). The fluctuation amplitude in the thickness of the ciliary muscle (ΔCMT), i.e. the ratio of indicators with a narrow and wide pupil, was also evaluated.

**Results:**

The ciliary muscle thickness of the amblyopic eye in group 1 was 808±38 μm for CMT_max_, 724±54 μm for CMT_1_, 446±44 μm for CMT_2_, and 223±37 μm for CMT_3_, these indicators in group 2 were 812±33, 735±33, 432±35, and 229±29 μm, respectively.

Children of group 1 have been found to have an increase in ΔCMT of the amblyopic eye. The value of ACMT_max_ increased from 21±6 to 30±4 μm, ACMT_1_ from 19±6 to 29±5 μm, ACMT_2_ from 12±4 to 16±4 μm, ACMT_3_ from 11±4 to 16±4 μm, which is associated with an increase in visual acuity and a decrease in the refractive component. All changes within the group were statistically significant (p<0.01).

**Conclusion:**

OCT is a fairly informative method for studying the accommodative structures of the eye in children, providing the opportunity to objectively assess the amplitude of fluctuations in the thickness of the ciliary muscle during the treatment. It has been established that after refraction operation, the work of the ciliary muscle of the amblyopic eye was significantly improved, which is reflected in the increased values of ΔCMT, CMT_2_, and CMT_3_ and brings these parameters closer to those of the better paired leading eye.

## Introduction

Exploration of the accommodative apparatus biomechanics is necessary for the development of new approaches to the restoration of the accommodative ability of the eye [1]. One of the challenging problems is ciliary muscle imaging and understanding its interaction with the lens during accommodation *in vivo* [2].

The first information about physiological functioning of the ciliary muscle in static conditions was obtained from the results of the postmortem histological examinations of the rhesus monkeys having the accommodative structure of the eyes similar to that of humans [3]. It has been previously reported on the differences in the morphology of the ciliary muscle of the young and adult eyes [4-6] although the effect of these differences on the muscle functioning has not been shown. Images of the lens and/or ciliary muscle in static accommodation conditions were acquired using magnetic resonance imaging (MRI), ultrasound, Scheimpflug camera, ultrasound biomicroscopy (UBM), and time-domain optical coherence tomography (OCT) at the wavelengths of about 1300 nm [5, 6]. MRI produces distortion-free images, but a low velocity of image production limits its application for the study of dynamic accommodation. UBM application is also restricted due to the contact of the probe with the ocular surface and usage of anesthetics during investigations. These manipulations may cause psychological discomfort and sometimes allergic reactions [7]. Besides, MRI and UBM are performed in prone position, which reduces the validity of the results obtained. To diagnose accommodation in children, it is preferable to use fast non-invasive procedures with maintaining the original position, therefore, the OCT technique seems to be the most optimal modality.

The ciliary muscle is known to participate in accommodation and possibly influence emmetropization, however, there are relatively scanty investigations of the ciliary muscle *in vivo.*

**The aim** is to study the state of the ocular accommodative apparatus in children with hyperopic anisometropia and amblyopia in dynamics.

## Materials and Methods

The study included 60 patients at the age of 6 to 15 years with anisometropia over 3 D, high and moderate degree of amblyopia, and with a spherical equivalent refraction (SE) from +3.5 to +7.25 D on the amblyopic eye. The study was carried out in compliance with the Declaration of Helsinki developed by the World Medical Association (2013) and Federal Law of the Russian Federation No.323-FZ of November 21, 2011 “On the fundamentals of public health protection in the Russian Federation”. The study was approved by the Ethical Committee of the Cheboksary Branch of S. Fyodorov Eye Microsurgery Federal State Institution of the Ministry of Health of Russia (Cheboksary, Russia). Written informed consent was obtained from the children’s parents to the ophthalmologic examination, treatment, and usage of the data for scientific purposes.

Patients were divided into two groups (n=30 in each). Group 1 included children who were medically indicated to undergo femtosecond laser intrastromal *in situ* keratomileusis (FS-LASIK) [8, 9] on the amblyopic eye. Group 2 consisted of the children with a comparable initial status wearing spectacles. During 2 years, all patients received device-assisted treatment for amblyopia (laser-, magneto-, photo-, and electrostimulation) 2 times a year. A general description of patients is presented in Table 1. In our study, amblyopia occurred on the left eye more often, high degree hyperopia (83.3% in group 1 and 46.7% in group 2) and high degree amblyopia (70.0% in group 1 and 56.7% in group 2) prevailed.

**T a b l e 1.** Description of the patients (n/%)

Parameters	Children after FS-LASIK (group 1)	Children wearing spectacles (group 2)
Gender:		
female	13/43.3	14/46.7
Male	17/56.7	16/53.3
Amblyopic eye:		
Right	9/30.0	10/33.3
Left	21/70.0	20/66.7
Moderate hyperopia	5/16.7	16/53.3
High hyperopia	25/83.3	14/46.7
Moderate amblyopia	9/30.0	13/43.3
High amblyopia	21/70.0	17/56.7

A standard ophthalmological diagnostic procedure has been carried out: refractometry before and after cycloplegia, uncorrected vision acuity (UCVA) and best-corrected visual acuity (BCVA) tests in decimal units or using LogMAR units according to Holladay’s formula [10] for 5 m and 50 cm.

**Figure 1.**
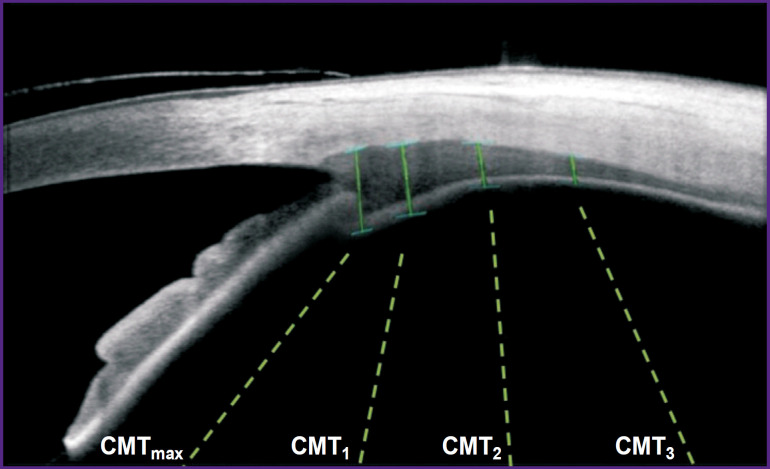
Examination of the temporal side of the ciliary muscle on the optical coherence tomography system CASIA2

The parameters of the eye accommodative structures were additionally evaluated on the optical coherence tomographic system CASIA2 (Tomey, Japan) under natural accommodation with a narrow pupil — with a preliminarily placed contact lens having an optical power equal to the best vision acuity and with medicamentally inhibited accommodation after the removal of the contact lens. The temporal part of the ciliary muscle was examined since it is more readily accessible for imaging and analysis. When capturing the images of the temporal part of the ciliary muscle, a black snowflake was used as a target on a white background having the size corresponding to the vision acuity of 0.1 at a 33-cm distance. The gaze was displaced towards the nose by 40°. This angle allows fixing the target at a minimum turn of the eye, when the optical axis of the scanner passes through the sclera rather than through the cornea, which reduces optical distortions. Ciliary muscle thickness (CMT) was analyzed at four levels relative to the scleral spur: maximum thickness of the ciliary muscle CMT_max_, and CMT!, CMT_2_, CMT_3_ were taken from the scleral spur at a distance of 1,2, and 3 mm, respectively (Figure 1).

**Figure 2.**
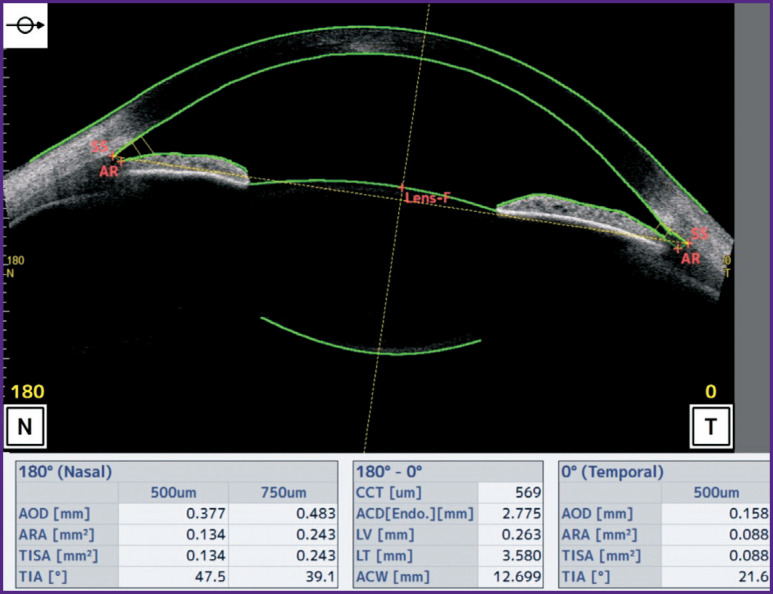
Examination of the anterior-posterior size of the crystalline lens and anterior chamber on the optical coherence tomography system CASIA2

Anterior-posterior size of the anterior chamber and the crystalline lens (Figure 2) was measured in a way similar to the examination of the ciliary muscle, first with a narrow pupil (the gaze was fixed directly on the target) and then under cycloplegia. Three measurements were done, and the average values were calculated.

**Statistical data** were processed using the following software: Statistica 10.0 (StatSoft, USA) and Microsoft Office Excel (Microsoft, USA). Variables were checked for the normality of distribution using Kolmogorov-Smirnov test. Traditional indicators of descriptive statistics were used in the study: the number of observations (n), arithmetic mean (M), standard deviation (SD), and categorical data (%). The Student’s t-test for dependent and independent samples was used to compare data before and after the operation. Differences between the sample values were considered significant at p<0.05. Equality of variances was tested using Fisher’s F-criterion.

## Results

Before the amblyopic eye treatment, SE measured with a wide eye was equal to +6.77±1.80 D, anisometropia was +4.25± 1.40 D in children of group 1. In group 2, these values were within the range of +5.9±2.5 and 4.4±1.9 D, respectively. Refraction of the better eye was close to emmetropia. BCVA values for 5 m were comparable between the groups prior to the treatment and amounted to 0.12±0.08 (LogMAR — +0.90±0.31) in group 1 and 0.19±0.17 (LogMAR — +0.72±0.42) in group 2.

**Figure 3.**
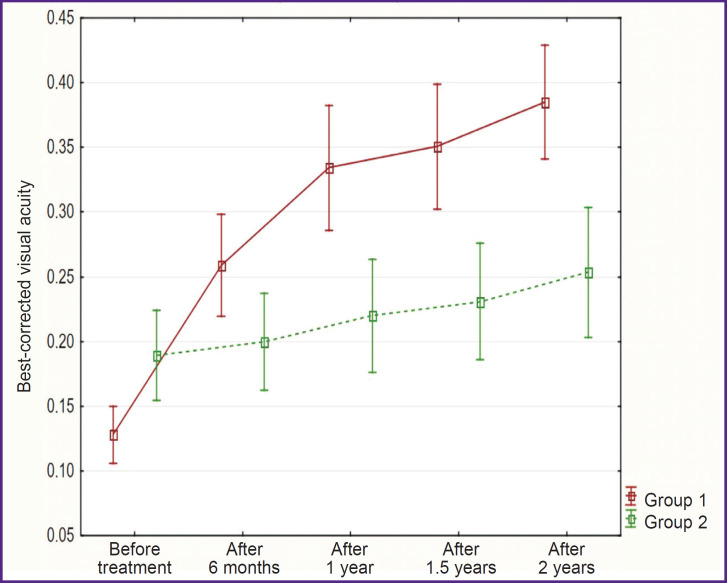
The change in best-corrected visual acuity during the conducted treatment in the groups

After 2 years of treatment, an average BCVA value at a 5 m distance was equal to 0.40±0.09 (LogMAR — +0.40±0.16) in group 1 and 0.25±0.10 (LogMAR — +0.60±0.13) in group 2 (p=0.01) (Figure 3).

Spherical equivalent refraction of the operated amblyopic eye in group 1 at the end of the follow-up period decreased to +1.31 ±0.16 D (p<0.001 relative to the value before the treatment), while the degree of anisometropia decreased to +1.29±1.10 D (p<0.001). These values remained at the initial level in group 2. By the end of the follow-up period, the differences between the two groups were statistically significant (p<0.001).

The increase of the BCVA value at a 50-cm distance has been noted in both groups after the conducted treatment. In group 1, BCVA increased by 0.21±0.09 and was equal to 0.32±0.12; in group 2, the increase was by 0.05±0.05 and made up 0.16±0.12; the data between the groups were statistically significant (p=0.02).

During the entire follow-up period, the gain in the ciliary muscle thickness was noted (Tables 2 and 3). In group 2, the thickness increase in the original parts of the ciliary muscle of the amblyopic eye was more prominent. By the end of the treatment, the differences between the groups at the level of CMT_max_ and CMT_1_ were statistically significant (p=0.04 and p=0.05, respectively). In the posterior parts, vice versa, the greater gain was noted in group 1. After 2 years, the differences between the groups were statistically significant (p=0.04) at the CMT_2_ level. The differences between the groups in the paired leading eye were statistically significant at the level of CMT_max_ (p=0.04) at the end of the follow-up.

**T a b l e 2.** The level of increase in the ciliary muscle thickness of the amblyopic eye according to the OCT data (μm), M±SD

Parameters	Before treatment	After 6 months	After 1 year	After 1.5 years	After 2 years	p*
CMT_max_:						
group 1	808±38	813±38	821±34	824±33	829±33	0.07
group 2	812±33	818±38	830±38	836±37	840±36	**0.02**
p_1-2_	0.11				**0.04**	
CMT_1_:						
group 1	724±54	729±54	733±53	740±53	744±52	**0.05**
group 2	735±33	746±34	752±33	759±24	761±29	**0.03**
p_1-2_	0.07				**0.05**	
CMT_2_:						
group 1	446±44	451±31	458±30	461±31	465±30	**0.03**
group 2	432±35	435±38	436±39	439±38	441±36	0.13
p_1-2_	0.06				**0.04**	
CMT_3_:						
group 1	223±37	229±33	234±31	237±32	241±17	**0.05**
group 2	229±29	231±26	233±28	234±29	236±25	0.25
p_1-2_	0.23				0.09	

* the level of significance for the results compared before treatment and after 2 years.

**T a b l e 3.** The level of increase in the ciliary muscle thickness of the leading eye according to OCT data (μm), M±SD

Parameters	Before treatment	After 6 months	After 1 year	After 1.5 years	After 2 years	p*
CMT_max_:						
group 1	787±38	790±37	794±41	798±38	803±39	0.12
group 2	797±39	802±40	812±38	819±42	823±43	0.07
p_1-2_	0.16				**0.04**	
CMT_1_:						
group 1	694±46	699±45	705±45	708±44	712±45	0.08
group 2	701±46	710±51	718±49	722±50	727±48	0.06
p_1-2_	0.25				0.06	
CMT_2_:						
group 1	459±30	462±31	465±29	469±30	472±31	0.07
group 2	453±31	456±30	460±29	462±28	463±26	0.10
p_1-2_	0.21				0.1	
CMT_3_:						
group 1	248±38	251±40	253±35	253±38	255±40	0.09
group 2	242±31	243±30	245±28	247±25	247±30	0.18
p_1-2_	0.33				0.12	

* the level of significance for the results compared before treatment and after 2 years.

Functional changes of accommodation were evaluated by the amplitude of thickness fluctuation of the ciliary muscle (ΔCMT), representing the ratio of ciliary muscle thickness, taken at the medicamentally inhibited accommodation, to the ciliary muscle thickness, taken before dropping cycloplegics with target fixation.

**Figure 4.**
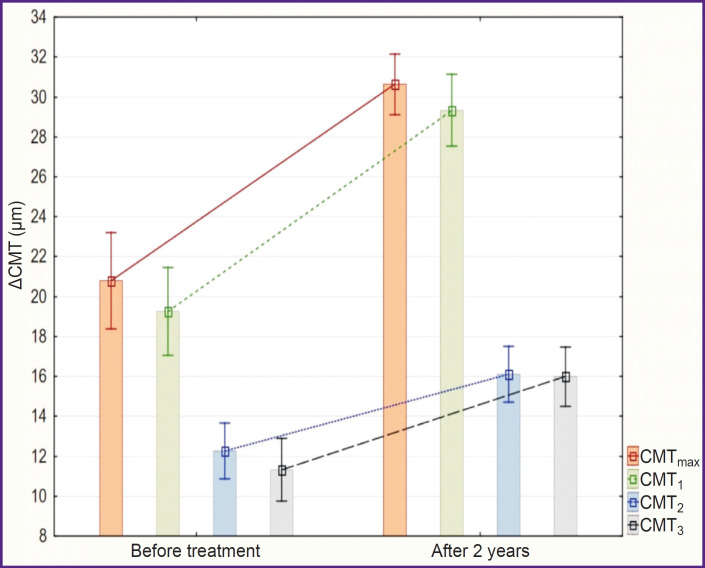
Changes in ΔCMT of the amblyopic eye during the conducted treatment in the group of children after FS-LASIK (M±SD)

At the end of the follow-up period, the ΔCMT_max_ value of the amblyopic eye increased in group 1 from 21±6 to 30±4 μm, ΔCMT-i — from 19±6 to 29±5 μm, ACMT_2_ — from 12±4 to 16±4 μm, ACMT_3_ — from 11±4 to 16±4 μm (Figure 4). All intragroup changes were statistically significant (p<0.01).

**Figure 5.**
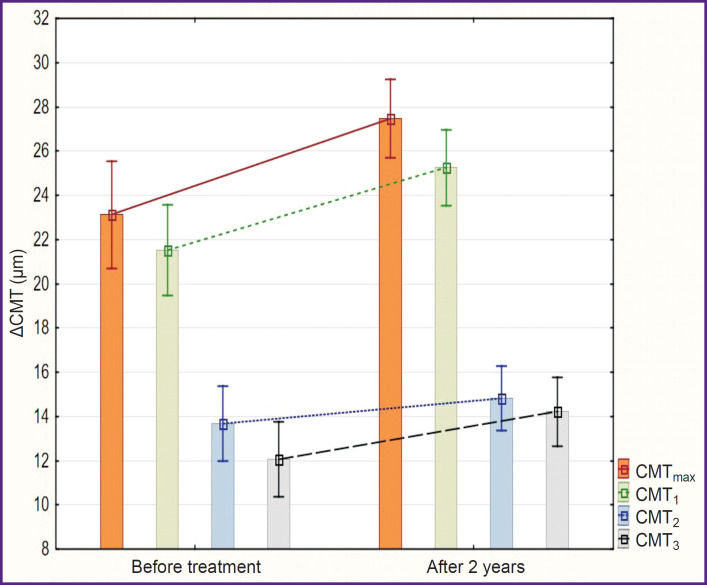
Changes in ΔCMT of the amblyopic eye during the conducted treatment in the group of children with spectacle correction (M±SD)

Increase of ΔCMT was also noted in group 2. The ΔCMT_max_ value before treatment was 23±5 μm, ΔCMT! — 22±6 μm, ACMT_2_ — 14±5 μm, ACMT_3_ — 12±5 μm. After 2 years, the value of ΔCMT_max_ was 27±4 μm (p=0.005 relative to the value before the treatment), ΔCMT! — 25±5 μm (p=0.003), ACMT_2_ — 15±4 μm (p=0.13), ACMT_3_ — 14±5 μm (p=0.02) (Figure 5). Changes within group 2 were statistically significant but less marked than in group 1.

**Figure 6.**
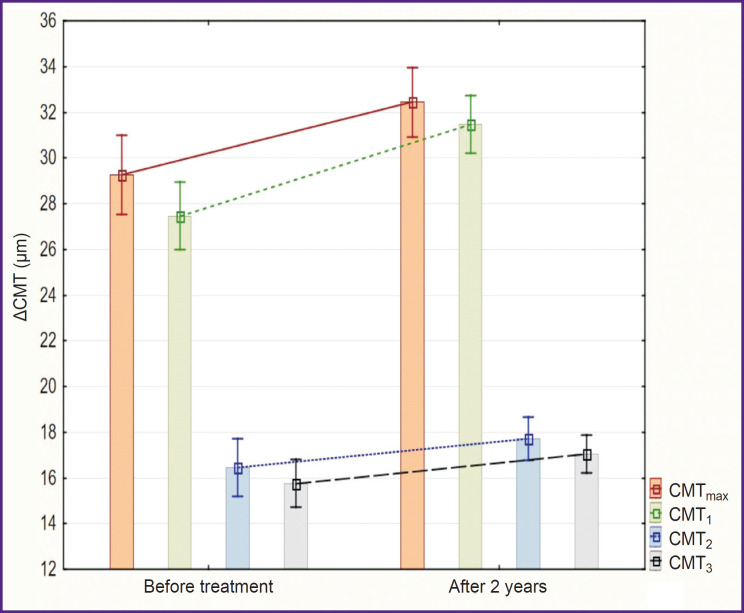
Changes in ΔCMT of the leading eye during the conducted treatment in the group of children after FS-LASIK (M±SD)

**Figure 7.**
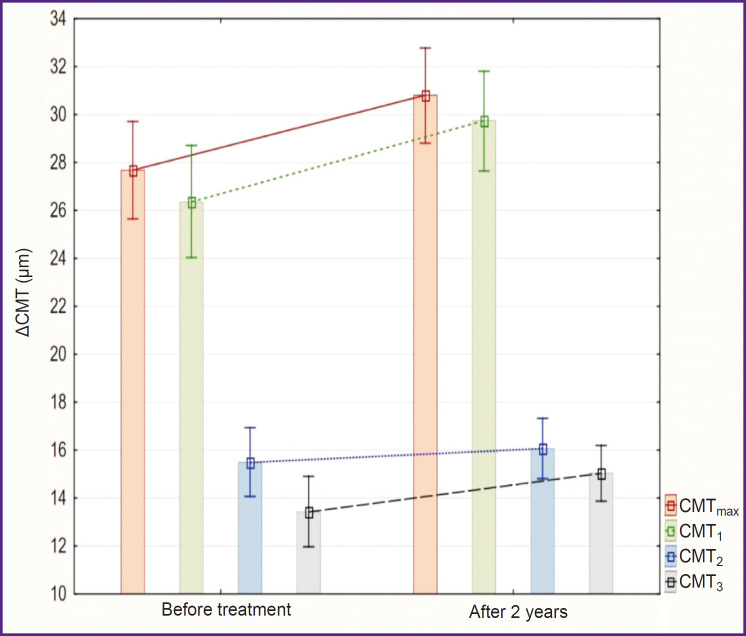
Changes in ΔCMT of the leading eye during the conducted treatment in the group of children with spectacle correction (M±SD)

In both groups, there was increase in ΔCMT at all levels in a paired leading eye and in amblyopic eye as well. In group 1, the ACMT_max_ value increased from 29±5 to 32±4 μm (p=0.005), ACMT_1_ from 27±4 to 31±4 μm (p=0.001), ΔCMT_2_ from 16±4 to 18±3 μm (p<0.001), ΔCMT_3_ from 16±3 to 17±3 μm (p=0.05) (Figure 6). In group 2, the ΔCMT_max_ value increased from 27±5 to 31±5 μm (p=0.01), ΔCMT_1_ from 26±6 to 29±5 μm (p<0.001), ΔCMT_2_ from 15±4 to 16±3 μm (p=0.06), ΔCMT_3_ from 13±4 to 15±3 μm (p=0.02) (Figure 7). Two years after the treatment, differences of ΔCMT_1_ values between the groups were statistically significant (p=0.05).

When investigating the lens thickness and the depth of the anterior chamber of the amblyopic eye with a narrow and wide pupil, no statistically significant changes before and after the treatment have been found. In group 1, the lens thickness with a narrow pupil before treatment was 3.40±0.25 mm, with a wide pupil 3.30±0.20 mm; in group 2 the values were 3.30±0.15 and 3.30±0.22, respectively. After 2 years, the indicators between the groups were also close. Thus, in group 1, the crystalline lens thickness with turned-on accommodation was 3.40±0.16, with turned-off accommodation it was equal to 3.20±0.18; in group 2 it was 3.40±0.19 mm (p=0.21 relative to group 1) and 3.30±0.15 (p=0.43), respectively. The depth of the anterior chamber in children after FS-LASIK with turned-on accommodation was 2.96±0.17 mm at the beginning of the study, with turned-off accommodation it was 3.10±0.20 mm; in the group of children with spectacle correction these values were 3.01±0.18 and 3.11 ±0.23 mm, respectively. After the treatment, these indicators were at the level of 3.09±0.20 and 3.14±0.16 mm in group 1; and 3.10±0.21 mm (p=0.18 relative to group 1) and 3.12±0.22 mm (p=0.34), respectively, in group 2. The values of the lens thickness and anterior chamber for the paired leading eye were similar to those for the amblyopic eye.

## Discussion

Amblyopia is a cause of refraction disability. Anisometropia occurs in 35-45% of patients with hyperopia. In hyperopic amblyopia, anisometropia is encountered in 89.3-96.3% of cases [11]. A late diagnosis of this problem makes it difficult to restore visual functions. Analyzing amblyopia of various intensity degree in children with severe refraction disorders, it has been established that accommodation is a leading function involved in the development of vision acuity [12]. Thus, the effectiveness of amblyopia treatment depends on the severity of accommodative disorders.

The comparison of mean values of the amblyopic and paired eye in both groups has demonstrated that before the treatment, the ciliary muscle of the leading eye was thinner by 19 μm at the CMT_max_ level and by 32 μm at the CMT! level in the anterior parts, in the depth of the posterior parts it was thicker by 17 μm at the CMT_2_ level and by 19 μm at the CMT_3_ level. The increase in the posterior compartments was higher in children after FS-LASIK, i.e. by 19 μm at the CMT_2_ level and by 18 μm at the CMT_3_ level, in children with spectacle correction the values of these indicators increased by 9 and 7 μm, respectively. On the contrary, a large increase in the thickness of the anterior part of the ciliary muscle was observed in children of group 2 by 28 μm at the CMT_max_ level and by 26 μm at the CMT_1_ level. In group 1, these values increased by 21 and 20 μm, respectively.

The diagnostic examination of children with anisometropia using OCT was conducted by Lewis et al. [13]. According to the authors’ data, the ciliary muscle thickness was 756.7 μm at the CMT_max_ level, 730 μm at the CMT_1_ level, 525 μm at the CMT_2_ level, and 315 μm at the level of CMT_3_. The data of the present study are close to these indicators, however, we registered higher parameters (806 and 724 μm) in the anterior parts of the ciliary muscle, which is probably determined by the presence of amblyopia and a higher degree hyperopia in the examined children.

In our study, the increase in the fluctuation amplitude of the ciliary muscle thickness (ΔCMT) for the amblyopic eye has been revealed in children after FS-LASIK, which seems to be associated with the improvement of visual acuity and significant reduction of the refraction component.

The biometric data on the eye without cycloplegia and with it were studied by Tarutta et al. [14]. It has been shown by the OCT data that at a mean refraction component of +3.5±1.2 D, the depth of the anterior chamber without cycloplegia was 3.49±0.02 mm, with cycloplegia — 3.63±0.02 mm; the thickness of the crystalline lens without cycloplegia was 3.60±0.03 mm, with cycloplegia — 3.50±0.03 mm, which agrees with the results obtained by us.

## Conclusion

Optical coherence tomography is a sufficiently informative method of investigation of ocular accommodative structures in children providing objective assessment of the amplitude of fluctuation of the ciliary muscle thickness during the conducted treatment. Monitoring of the accommodation structures has shown that after the refraction operation, in addition to the reduction of the refractive disorder and enhancement of visual functions, there was a significant improvement in the work of the ciliary muscle of the amblyopic eye, which is evidenced by the increase in the ΔCMT, CMT_2_, and CMT_3_ indicators, bringing these parameters closer to the better paired leading eye.

## References

[1] Charman W.N. Developments in the correction of presbyopia II: surgical approaches. Ophthalmic Physiol Opt 2014; 34(4): 397-426, 10.1111/opo.12129. 24716827

[2] Koshitz I.N. Svetlova O.V. Egemberdiev M.B. Guseva M.G. Traditional and new mechanisms of accommodation and their classification. Rossijskaa detskaa oftal’mologia 2018; 3: 20-36.

[3] Neider M.W. Crawford K. Kaufman P.L. Bito L.Z. In vivo videography of the rhesus monkey accommodative apparatus: age-related loss of ciliary muscle response to central stimulation. Arch Ophthalmol 1990; 108(1): 69-74, 10.1001/archopht.1990.01070030075032. 2297335

[4] Strakhov V.V. Klimova O.N. Korchagin N.V. The clinical picture of active accommodation for far vision. Rossijskij oftal’mologiceskij zurnal 2018; 11(1): 42-51, 10.21516/2072-0076-2018-11-1-42-51.

[5] Monsalvez-Romln D. Dominguez-Vicent A. Esteve-Taboada J.J. Montés-Mico R. Ferrer-Blasco T. Multisectorial changes in the ciliary muscle during accommodation measured with high-resolution optical coherence tomography. Arq Bras Oftalmol 2019; 82(3): 207-213, 10.5935/0004-2749.20190041. 30810617

[6] Laughton D.S. Coldrick B.J. Sheppard A.L. Davies L.N. A program to analyse optical coherence tomography images of the ciliary muscle. Cont Lens Anterior Eye 2015; 38(6): 402-408, 10.1016/j.clae.2015.05.007. 26072268

[7] Kao C.Y. Richdale K. Sinnott L.T. Grillott L.E. Bailey M.D. Semiautomatic extraction algorithm for images of the ciliary muscle. Optom Vis Sci 2011; 88(2): 275-289, 10.1097/opx.0b013e3182044b94. 21169877 PMC3030281

[8] Alio J.L. Wolter N.V. Pinero D.P. Amparo F. Sari E.S. Cankaya C. Laria C. Pediatric refractive surgery and its role in the treatment of amblyopia: meta-analysis of the peer-reviewed literature. J Refract Surg 2011; 27(5): 364-374, 10.3928/1081597x-20100831-01. 20839663

[9] Paysse E.A. Tychsen L. Stahl E. Pediatric refractive surgery: corneal and intraocular techniques and beyond. J AAPOS 2012; 16(3): 291-297, 10.1016/j.jaapos.2012.01.012. 22681949

[10] Holladay J.T. Proper method for calculating average visual acuity. J Refract Surg 1997; 13(4): 388-391, 10.3928/1081-597x-19970701-16. 9268940

[11] Apaev A.V. Tarutta E.P. Comparative assessment of the parameters of visual fixation in amblyopia of different origin. Vestnik oftal’mologii 2020; 136(2): 26-31, 10.17116/oftalma202013602126. 32366066

[12] Toor S. Horwood A.M. Riddell P. Asymmetrical accommodation in hyperopic anisometropic amblyopia. Br J Ophthalmol 2018; 102(6): 772-778, 10.1136/bjophthalmol-2017-310282. 29051327 PMC5787021

[13] Lewis H.A. Kao C.Y. Sinnott L.T. Bailey M.D. Changes in ciliary muscle thickness during accommodation in children. Optom Vis Sci 2012; 89(5): 727-737, 10.1097/opx.0b013e318253de7e. 22504329 PMC3348375

[14] Tarutta E.P. Harutyunyan S.G. Milash S.V. Khandzhyan A.T. Khodzhabekyan N.V. Proskurina O.V. Change in the ophthalmobiometric parameters in myopia and hyperopia under the influence of cycloplegia. Oftal’mologia 2018; 15(1): 58-63, 10.18008/1816-5095-2018-1-58-63.

